# Incorporation of graphene oxide in polyethersulfone mixed matrix membranes to enhance hemodialysis membrane performance†

**DOI:** 10.1039/c7ra11247e

**Published:** 2018-01-03

**Authors:** M. Z. Fahmi, M. Wathoniyyah, M. Khasanah, Y. Rahardjo, S. Wafiroh, Abdulloh

**Affiliations:** Department of Chemistry, Universitas Airlangga Surabaya 61115 Indonesia m.zakki.fahmi@fst.unair.ac.id +62-31-5922427 +62-31-5922427; Institute of Tropical Disease, Airlangga University Surabaya 61115 Indonesia

## Abstract

Graphene is a carbon allotrope and possesses numerous unique properties which make it an attractive material in many areas. In this work, graphene oxide (GO) was added to polyethersulfone (PES) mixed matrix membranes (MMMs) to improve the performance of hemodialysis membranes. GO was synthesized from tartaric acid by pyrolysis with various temperatures of the pyrolysis and the membrane was fabricated by a casting solution method followed by its characterization. The MMMs showed better mechanical properties than pristine PES with a tensile stress and tensile strain value of 5.55 MPa and 0.039 m, respectively. The hydrophilicity of the membranes which is in agreement with contact angle values showed that GO addition increased the hydrophilicity of the MMMs. Hence, the solute flux and clearance of creatinine gave values of 2.94 L m^−2^ h^−1^ and 78.3%, respectively. Cross sectional images and the surface morphology were also recorded using scanning electron microscopy (SEM). The resulting data proved that the modified MMMs can be a potential material for hemodialysis.

## Abstract 2

In this work, graphene oxide (GO) was added to polyethersulfone (PES) mixed matrix membranes (MMMs) to improve the performance of hemodialysis membranes.

## Graphical abstract

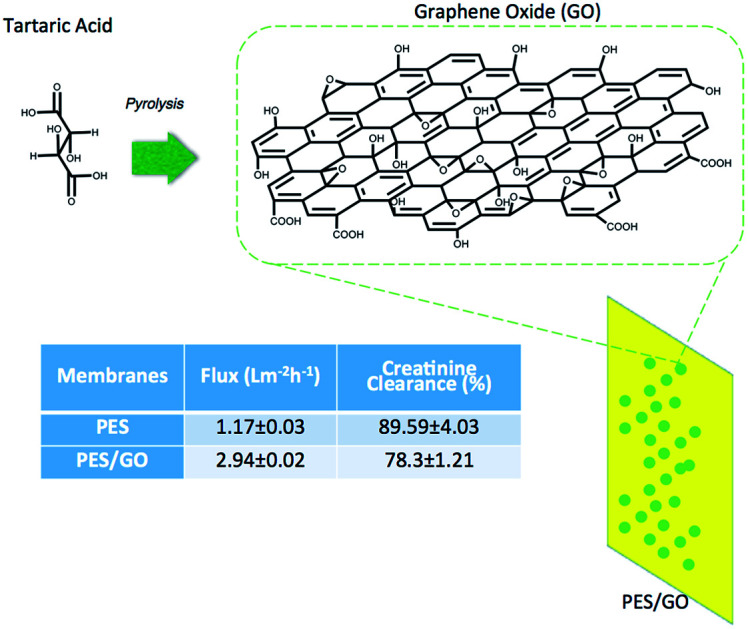

## Introduction

Chronic kidney disease (CKD) is a world-wide disease and was ranked 17^th^ as the leading cause of death globally based on a study by the Global Burden of Disease (GBD) in 2015.^1^ The Kidney Disease Quality Outcome Initiative (KDQOI) defined CKD as kidney damage or a Glomerulus Filtration Rate (GFR) < 60 mL min^−1^/1.73 m^2^ for 3 months or more regardless of the cause.^2^ Kidney damage will occur with increasing concentration of metabolic waste, such as creatinine and urea, and the effects will lead to other harmful health conditions.^3^ Therefore, CKD patients require supportive therapies to prolong life such as hemodialysis (HD), peritoneal dialysis, or kidney transplant.^4^ The main component of hemodialysis treatment is the membrane which is used as a barrier to remove metabolic waste from blood.^5^ Hemodialysis membranes have been made from various polymers. Among several polymers proposed, polyethersulfone (PES) is widely used for blood purification due to its outstanding hydrolytic, oxidative, and thermal stability, and excellent mechanical properties.^6^ Application of PES as the main material of the membrane has also been considered due to its excellent properties for improving the performance and the fouling resistance of the membrane.^7^ However, the hydrophobic nature of PES may cause membrane fouling which is mainly caused by the deposition of solutes, such as blood protein, that adsorb rapidly on the surface of the membrane or into the membrane pores.^8^ Therefore, modification of PES is an important way to reduce this problem. Several hydrophilic modifications have been reported and revealed that this method can be applied to reduce the membrane’s fouling and prevent the decreasing of the membrane’s flux.^9–11^ The common modification method is by adding some hydrophilic polymer,^12^ but the result is not satisfactory because of the poor tolerance of the polymeric membrane to high temperature, oxidants, strong acid or alkaline reagents, and organic solvent.^13^ Recently, the development of membranes has been shifted to mixed matrix membranes (MMMs) that combine adsorption and diffusion in one membrane to remove uremic solutes in blood. MMMs have been reported to have adsorptive particles that are incorporated into the polymer matrix.^14^

Meanwhile, carbon based materials will always be an interesting area for researchers due to their unique properties, both chemical and physical. Several advantageous properties, such as high thermal/chemical stability, electrical conductivity, surface area, and good corrosion resistance, are attributed to carbon materials. Carbon materials provide a wide variety of structures and textures ranging from 0D to 3D, such as C-dots and graphene quantum dots (0D), carbon nanotubes (1D), graphene and graphene oxide (2D), and graphite (3D).^15^ These carbon materials are easy to process and are compatible with other materials due to their diverse surface chemical properties, making these materials ideal to be used in composites.

Some studies have been done using carbon materials in MMMs as the adsorptive material for hemodialysis membranes, such as activated carbon^16^ and multi walled carbon nanotubes.^12^ Another carbon material that has attracted much research in the field of separation membranes is graphene. Graphene is a 2D material that consists of a single layer of carbon arranged in an sp^2^ bonded aromatic structure. Graphene material has been confirmed as an excellent material to be used in the separation field due to its atomic thickness, high mechanical strength, and chemical inertness.^13^ The oxidized form of graphene is called graphene oxide (GO). In our previous work, GO has been successfully synthesized through pyrolyzing an appropriate precursor, such as citric acid.^17,18^ The direct pyrolyzing process of citric acid will obtain GQDs by tuning the degree of carbonization, while prolonged heating results in the complete carbonization of citric acid and GO is obtained.^19^

Incorporation of carbon nanotubes (CNTs) as an analogue of graphene with a PES matrix to improve the hydrophilicity of the membrane was proposed by Vatanpour *et al.*, in which some functional hydrophilic groups of the CNTs can attract the desired hydrophilic groups such as those of creatinine and uremic toxins and also be used as determining factors for the enhancement of the anti-fouling properties of the membrane.^20^ On the other hand, the hydrophobic nature of CNTs will provide hydrophobic sites to the membrane. Based on this report, application of GO to develop a method was based on the interactions in carbon nanotubes which have similar properties to those of GO.^12^ However, further improvement in the application of PES and GO for creatinine dialysis, the main part of the hemodialysis process, has not been achieved yet. In the present study, a PES/GO membrane was fabricated using the phase inversion method. The mechanical strength, functional group assessment, and hydrophilicity are important data that need to be improved as well as the morphology of the membrane. Finally, simulation of creatinine dialysis was performed using a dead-end system to examine the PES/GO membrane’s ability to remove creatinine, and it was compared with that of pristine PES to determine the influence of adding graphene into a PES matrix.

## Experimental section

### Materials

Tartaric acid (99.5%), sodium hydroxide (NaOH, 97%), picric acid (99%), and creatinine (98%) were purchased from Sigma-Aldrich (Milwaukee, USA). Polyethersulfone (PES) used as the membrane raw material was purchased from Raza Traders (Mumbai, India) and dimethylformamide (DMF) used as solvent was purchased from Merck & Co., Inc. (New Jersey, USA). All chemicals were used directly without further purification.

### Preparation of GO

GO was prepared from tartaric acid by the pyrolysis method. Experimentally, about five grams of tartaric acid was put in a glass container with a nitrogen flow and heated at various temperatures (270 °C, 300 °C, 350 °C, and 400 °C) with a heating rate of 30 °C per minute and the adjusted temperature was maintained for 3 hours. The resulting powder obtained from pyrolysis was GO and it was further characterized using Raman spectrometer and X-ray diffraction instruments.

### Fabrication of the PES/GO membrane

GO particles (0.5 mg) were dispersed in 100 mL of DMF as solvent with vigorous stirring for a minute, followed by ultra-sonication treatment (20 kHz, 130 W) to make the GO particles disperse completely. About 17 mg of PES was added and dissolved in the solution under continuous stirring until the polymer completely dissolved. This PES/GO solution was used for fabrication using the phase inversion method, where this dope solution was cast onto clean glass and rolled to form a flat-sheet membrane with a diameter of around 250 μm. The resulting membrane was subsequently put into a water containing coagulation bath for the coagulation process of the membrane without any evaporation process. The membrane was further stored in DI water for 24 hours and dried in between two pieces of filter paper at room temperature for 24 hours.

### Characterization of the membrane

FTIR analysis of the membrane was performed using a KBr pellet. The membrane was cut into small pieces and after being mashed with KBr was then analysed. For the tensile strength measurements, the membranes were cut into 6 × 1 cm sized pieces and their mechanical strength measured using an Autograph. The hydrophilic test was based on the contact angle of the membrane. The measurement was done using a contact angle goniometer. A 0.1 μL amount of water was dropped onto the membrane surface using a syringe. The contact angle was then measured per minute for 5 minutes. The morphology of the membranes was observed using a Scanning Electron Microscopy instrument.

### Cross flow system

The clearance of creatinine was observed to evaluate the performance of the membranes. Dialysis simulation was performed using a cross-flow system. The feed solution was streamed through the membranes. One hundred ppm of creatinine solution (dissolved in water) was prepared to mimic blood solution. This solution was passed through the membranes (retentate) and recirculated back to the feed reservoir. Meanwhile, solution that passed through the membranes was called the permeate and further observed. The membrane area used in the cross-flow system was 14.62 cm^2^. Before starting the experiment, the membranes were compacted to avoid the compaction effect of membranes.

The concentration of the creatinine solution was adjusted based on a calibration curve and was measured using a colorimetric assay by first making a complex of creatinine with picric acid as a chromogenic agent following a previous study with some modification.^21^ Experimentally, about 2 mL of creatinine solution was mixed with 1 mL of picric acid in 1 M NaOH solution (26 mM) and 1 mL of pure water with stirring for 15 min. UV-vis spectroscopy was carried out to analyse the creatinine–picric acid complex absorbance at a wavelength of 510 nm, and the creatinine concentration in the sample was calculated according to a standard curve.

The flux and creatinine clearance parameters were determined to evaluate the performance of the proposed membranes. The creatinine clearance during the dialysis simulation was determined using eqn (1).1
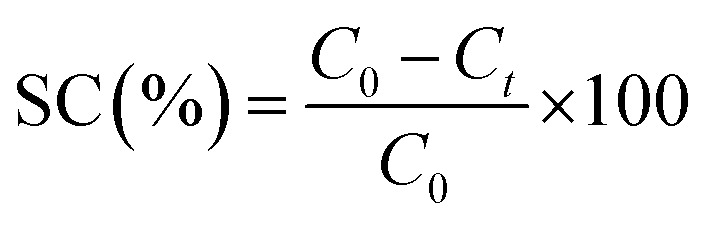
where SC (%) represents the solute clearance, and *C*_0_ and *C*_*t*_ are the creatinine concentration at an initial time before passing through the membrane and 40 minutes after passing through the membrane, respectively. Meanwhile, the flux was determined using eqn (2).2
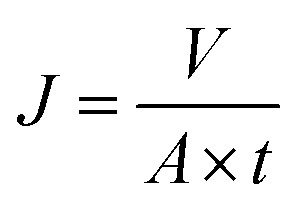
where *J* is the flux of the solute (L m^−2^ h^−1^), *V* is the volume of the diffuse solute (L), *A* is the diffusion area (m^−2^), and *t* is the diffusion time (hour).

## Results and discussion

### Synthesis and characterization of GO

**Scheme 1:**
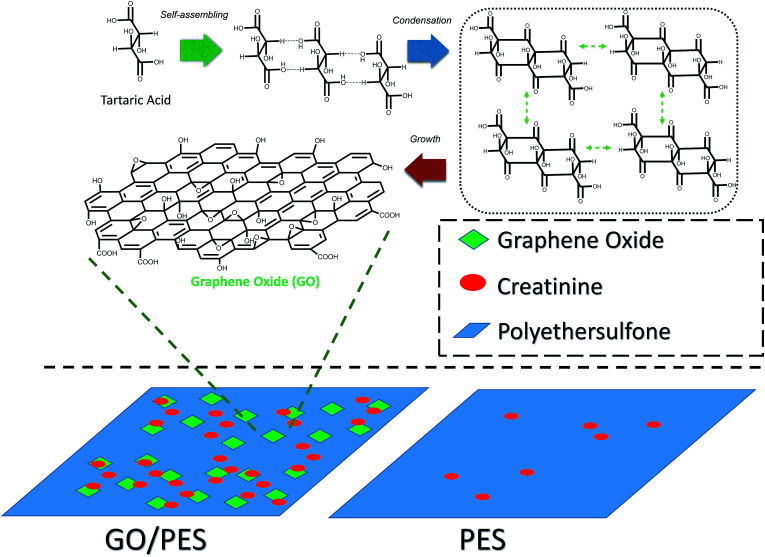
Schematic mechanism of GO formation.

GO was prepared from tartaric acid as the main precursor by the pyrolysis method. The precursor was heated at various temperatures. Close to its melting point, tartaric acid will be decomposed and release water molecules.^22^ This condition accelerates tartaric acid molecules to experience self-assembly once they have followed the condensation process to form six membered rings. This structure will grow and form the GO structure. A possible mechanism of GO formation from pyrolysis of tartaric acid is described in Scheme 1.

**Fig. 1:**
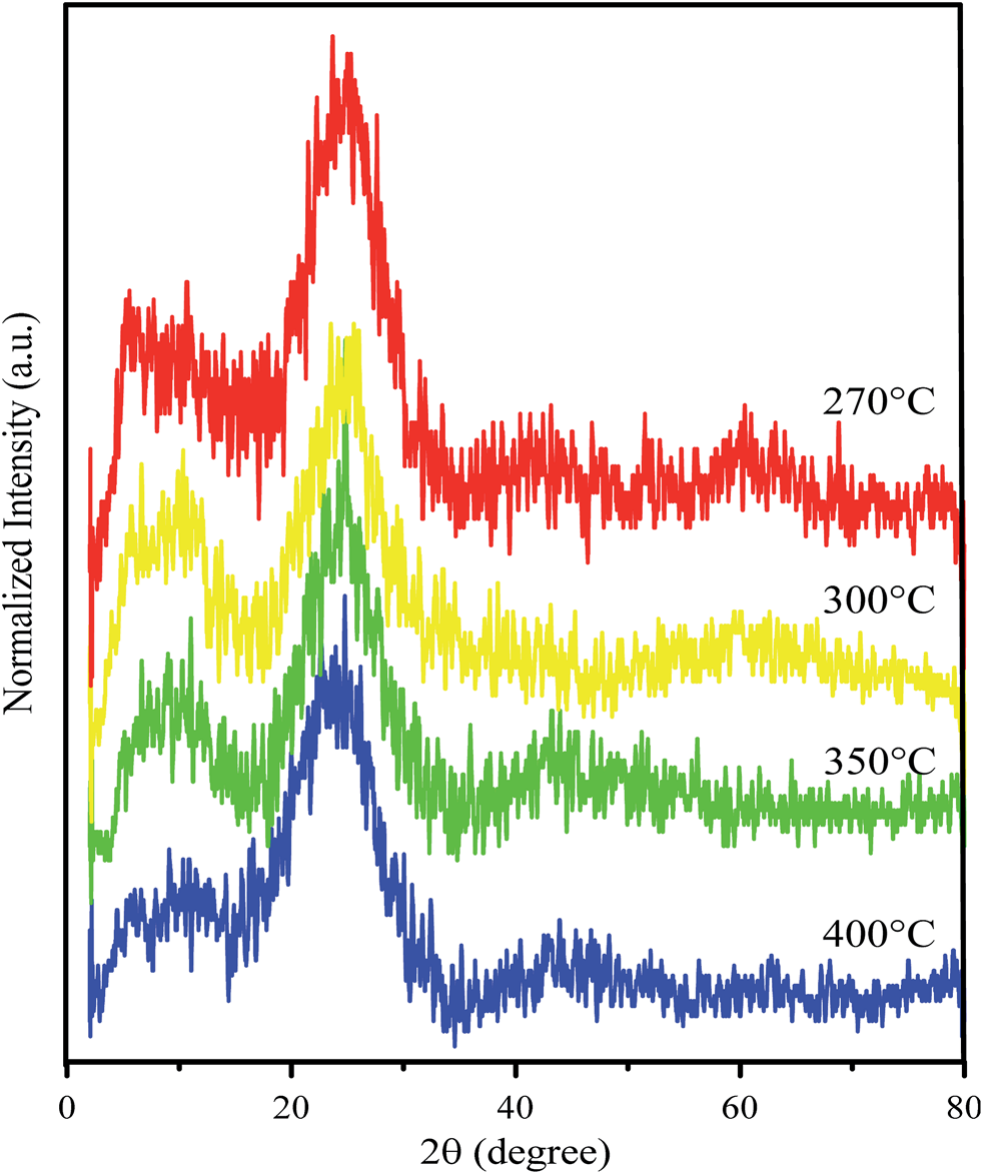
XRD patterns of the as prepared GO after being synthesized at various temperatures.

GO resulting from the pyrolysis process was further confirmed by Raman spectroscopy and X-ray diffraction (XRD) patterns. The diffraction pattern of GO shows two particularly broad peaks in the ranges 10–11° and 23–25° (Fig. 1). The broad peak that appeared at a 2*θ* of around 20–30° confirms a graphene structure with a *d*-spacing of about 3 Å.^17^ Meanwhile, formation of GO was confirmed by a peak which appeared at a 2*θ* of around 10–20° and a *d*-spacing value of around 7 Å.^20^ Compared with the *d* spacing of the graphene peaks, the left shifting of the GO *d*-spacing indicates a crystallinity change due to insertion of oxygen-containing groups attached at the edge and basal planes of GO. Interestingly, after improving the thermal variation for the synthesis process and assessing the peak intensities (Table 1), the GO area was observed to decrease following an increase of the synthesis temperature. The intensity ratio between the GO and graphene peaks significantly reveals that the graphene area should be much improved at high temperature. This phenomenon indicates a higher change in the crystallinity of GO due to the removal of oxygen-containing groups in the formed graphene.

**Table 1:** XRD intensity data of GO synthesized at various temperatures

Temperature of pyrolysis (°C)	Symbol	Peak GO/intensity 1 (a.u.)	Peak graphene/intensity 2 (a.u.)	Intensity ratio (*I*_2_/*I*_1_)
270	GO270	161	225	1.398
300	GO300	131	204	1.557
350	GO350	105	165	1.571
400	GO400	82	149	1.817

**Fig. 2:**
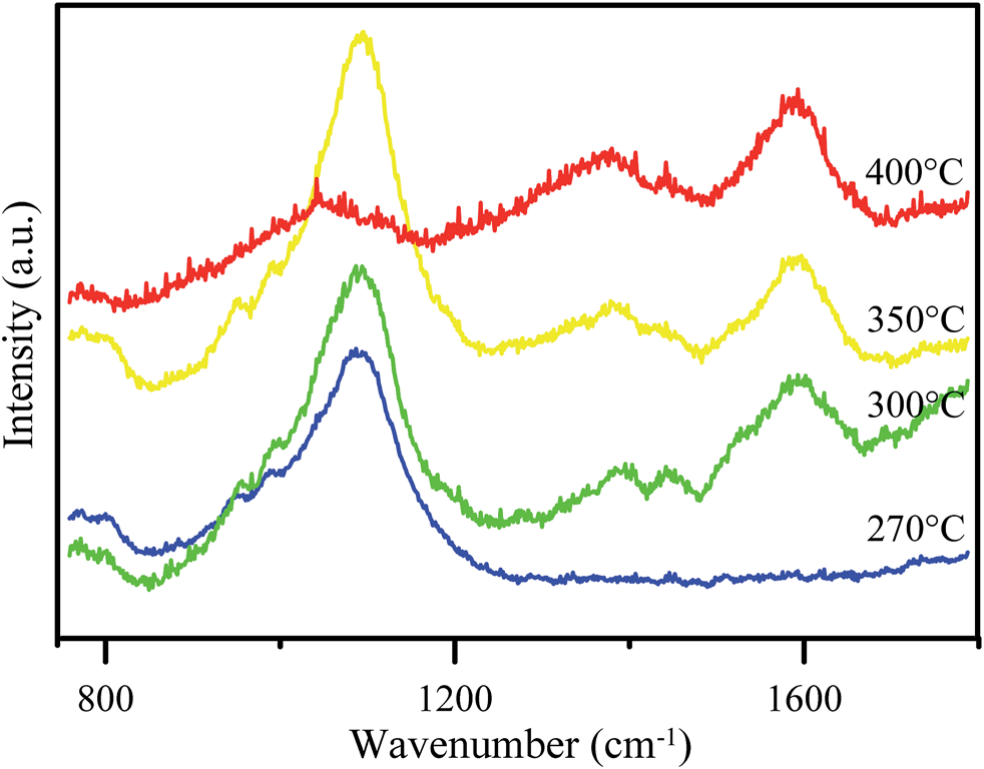
Raman spectra of GO after being synthesized at various temperatures.

The particular features of the graphene material in the Raman spectra are the bands which appeared at 1350 cm^−1^ and 1580 cm^−1^ corresponding to the D band and G band, respectively. The G peak which appeared corresponds to the bond stretching between all of the sp^2^ atoms in both the chains and ring. The D bands indicate sp^3^-hybridized carbon atoms.^23^Fig. 2 shows Raman spectra of GO synthesized at various temperatures. At 270 °C, the spectrum does not show either G or D bands and only shows a significant peak at ∼1100 cm^−1^ for C–O–C asymmetric bond stretching. This result shows that no GO or graphene was formed. Meanwhile, GO synthesized at higher temperature (300 °C, 350 °C, and 400 °C) showed both G and D bands. However, the D band for the 300 °C-synthesized GO was poor in intensity. A peak at ∼1100 cm^−1^ also appeared for all of the synthesized GO, but the poor intensity for the 400 °C-synthesized GO reveals an increasing graphene area instead of GO area.

The observed peaks in the Raman spectra indicate that the material which was synthesized in this research formed disordered graphene, which is also called graphene oxide (GO). This is because D bands only appear in disordered graphene.^24^ Besides, the peak at ∼1100 cm^−1^ showed that some epoxy groups are formed in the GO structure which can increase its hydrophilicity. Therefore, it can be used to increase the hydrophilicity of PES membranes.

## Characterization of membranes

### Functional groups of the GO-membrane

**Fig. 3:**
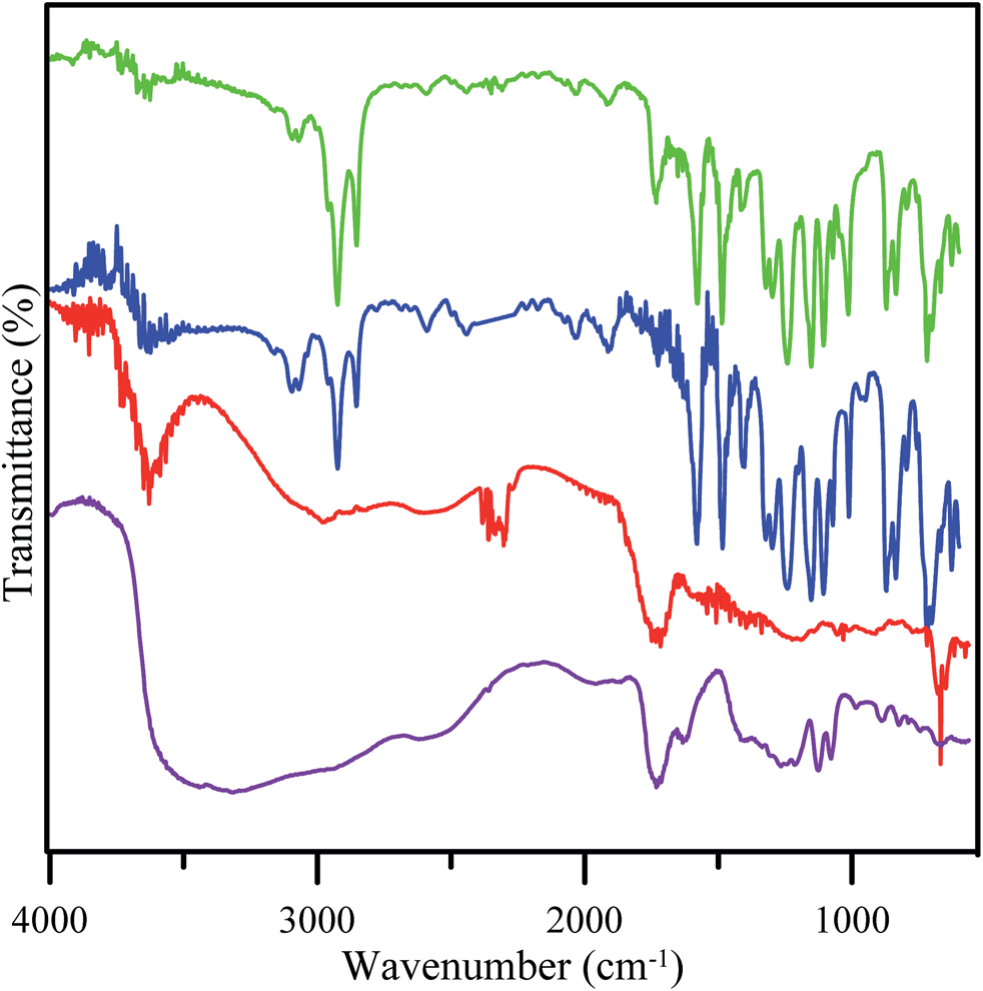
FTIR spectra of tartaric acid (purple line), GO350 (red line), the PES membrane (blue line), and the PES/GO membrane (green line).

The formation of GO from tartaric acid was confirmed by the decreasing intensity of the OH and carboxyl groups of tartaric acid (Fig. 3). This showed that the OH and carboxyl groups have been reduced to form GO.^21^ FTIR spectra of GO revealed the presence of an OH stretching vibration (3630 cm^−1^) and stretching vibrations for C=O (1718 cm^−1^) and C–OH (1219 cm^−1^) suggesting that oxygen containing groups are introduced into graphene oxide. This showed the disordered structure of GO. The figure also shows the FTIR spectra of the fabricated membrane. The result for both membranes of PES and PES/GO showed peaks for S=O asymmetric stretching at 1153.43 cm^−1^, C–O–C ether at 1107.14 cm^−1^, and C–H stretching at 2924.09 cm^−1^. Both membranes also showed peaks for C–C aromatic bonds but with different wavenumbers. The PES membranes showed a peak at 1579.70 cm^−1^ and the PES/GO membranes showed a peak at 1577.77 cm^−1^. Aromatic C–H also appeared at a different wavenumber, 3068.75 cm^−1^ and 3070.68 cm^−1^ for the PES and PES/GO membranes, respectively. The shift of the wavenumber for the aromatic group indicates the possibility of electrostatic bonding occurring. The possible bonding of PES/GO is π–π stacking due to the shift in the aromatic stretching frequencies. Meanwhile, incorporation of GO in the PES/GO membranes is confirmed by the peaks for OH at 3630.03 cm^−1^ and carboxylic C=O at 1732.08 cm^−1^ which were observed for both the GO and PES/GO membranes. Based on these XRD and FTIR data, GO350 was chosen mainly for the next process due to the presence of hydrophilic sites (such as hydroxyl or oxide sites) and hydrophobic sites (the graphene like structure) which were important for the design process.

### Membrane morphology

**Fig. 4:**
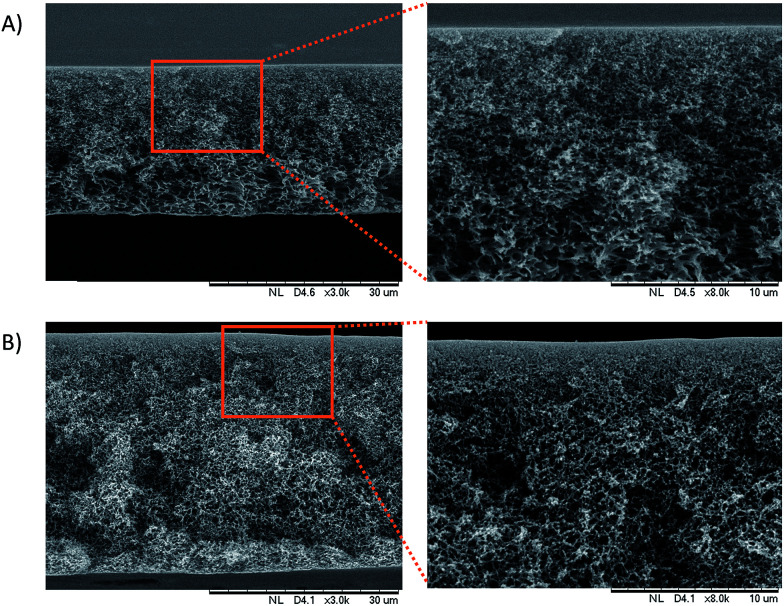
Cross sectional image of (A) the PES membrane and (B) the PES/GO350 membrane. The figures on the right show a higher magnification of a particular area (brown square).

**Fig. 5:**
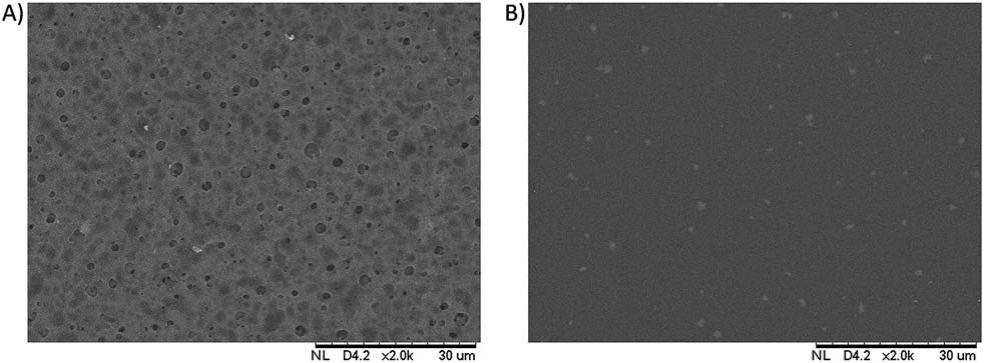
Surface morphology of (A) the PES membrane and (B) the PES/GO350 membrane.

The cross sectional morphologies of the PES/GO and PES membranes were analysed using SEM, as seen in Fig. 4. All of the membranes have an asymmetric structure composed of a dense skin layer and a compact spongy layer. The skin layer is responsible for permeation and solute rejection whereas the sponge sub-layer acts as the mechanical support of the membranes.^12^ The surface morphologies of the prepared membranes are shown in Fig. 5. The results show that the PES membranes had some inner pores whilst the PES/GO350 membranes showed a smooth surface. This can be related to the carbon-based structures of GO and the PES polymer, which caused the good dispersion of GO in the membrane matrix. This also showed that no agglomeration of nanoparticles was detected on the membrane surface confirming the uniform dispersion of GO into the membranes. Further EDX data (ESI, Fig. S1†) of the samples shown in Fig. 5 prove that the percentage of oxygen and carbon in PES/GO350 was higher than that in PES due to the addition of GO which consists of carbon and oxygen atoms. Beside these atoms, PES and PES/GO350 also contain sulphur atoms, where PES and PES/GO350 contain a sulphur percentage of 15.90% and 9.68%, respectively. The data also confirm that GO was uniformly dispersed in PES.

### Hydrophilicity evaluation

**Fig. 6:**
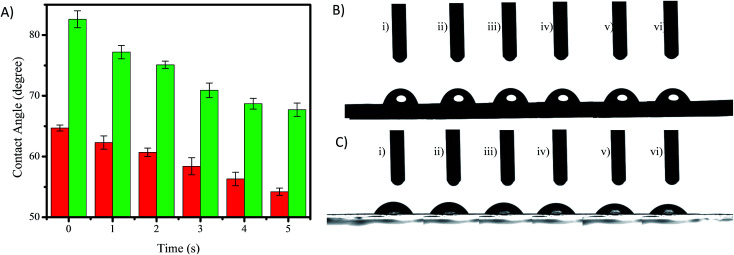
(A) The water contact angle values of the PES (red bars) and PES/GO350 (green bars) membranes, *n* = 3. Photographs of a water drop on PES (B) and PES/GO (C); the symbols (i) to (vi) represent the time at 0, 1, 2, 3, 4, and 5 s, respectively. The initial contact angle of PES (B-i) and PES/GO300 (C-i) was 82.09° and 64.71°, respectively.

Surface hydrophilicity is one of the important attributes to adjust the performance of hemodialysis membranes. Hydrophilic properties in membranes are good for hemodialysis membranes due to the membranes attracting blood protein to adsorb on the membrane’s surface and block the membrane pores.^12^ The hydrophilicity of a membrane can be evaluated by the water contact angle of a drop on the surface of the membrane, where a low contact angle confirms high water absorption and means high hydrophilicity of the membrane.^25^Fig. 6 shows the contact angles of the PES and PES/GO350 membranes, and shows that the contact angle of each membrane decreased with time due to a high water solubility. However, the contact angle of the membrane decreased drastically with the addition of GO350 into the membranes (PES/GO350), indicating that the hydrophilicity of the membranes increased with the addition of GO. The water contact angle of the PES and PES/GO350 membranes started off as 82.09° (Fig. 6(B-i)) and 64.71° (Fig. 6(C-i)), respectively. These contact angles decreased gradually with increasing time. The increased hydrophilicity with the addition of GO is mainly due to polar functional groups, such as epoxy, hydroxyl, and carboxylic groups, which are attached to GO and allowed better attraction of water molecules.

### Mechanical strength of the membranes

The mechanical strength of membranes also plays an important role in applying membranes because it represents their strength and compatibility for the hemodialysis process. The mechanical strength of membranes is expressed as the tensile stress and strain. The tensile stress and strain of the PES/GO350 membranes are 5.55 MPa and 0.039 m, respectively. Meanwhile, for unmodified PES they are 3.31 MPa and 0.033 m, respectively. The addition of GO350 into the membranes seems to improve the tensile stress of the membranes while not quite significantly improving the strain value. The Young’s modulus of the PES and PES/GO350 membranes was 10.2 × 10^−7^ and 16.9 × 10^−7^ Pa, respectively. This result showed that the PES/GO350 membranes have better mechanical properties than the PES membranes. The results of the mechanical properties of the membranes are shown in Table 2.

**Table 2:** Mechanical properties of the fabricated membranes (±SD, *n* = 3)

Membranes	Stress (MPa)	Strain (%)	Young’s modulus (MPa)
PES/GO270	4.22 ± 2.51	4.1 ± 0.021	11.4 ± 1.9
PES/GO300	3.23 ± 7.01	4.2 ± 0.013	8.1 ± 2.7
PES/GO350	5.55 ± 1.01	3.9 ± 0.004	16.9 ± 1.4
PES/GO400	6.25 ± 2.23	3.1 ± 0.005	20.8 ± 3.5
PES	3.31 ± 0.45	3.3 ± 0.007	10.2 ± 1.6

### Dialysis performance of the PES and PES/GO350 membranes

Clearance of creatinine was determined to assess the performance of the proposed membranes. The dialysis performance was evaluated *via* the cross flow method for the flat sheet membranes. In this case, the membranes were cut into circle shapes. The membrane’s performance was evaluated based on the clearance of creatinine from a feed reservoir as shown in Table 3.

**Table 3:** The results of the membrane’s dialysis performance (±SD, *n* = 3)

Membranes	Flux (L m^−2^ h^−1^)	Solute clearance (%)
PES	1.17 ± 0.03	89.59 ± 4.03
PES/GO270	3.11 ± 0.02	90.22 ± 5.12
PES/GO300	3.07 ± 0.15	81.34 ± 7.72
PES/GO350	2.94 ± 0.02	78.30 ± 1.21
PES/GO400	2.21 ± 0.07	85.56 ± 1.17

This table reveals that the solute flux of the PES/GO350 membranes is higher than that of the PES membranes, which can be related to increasing the membrane’s hydrophilicity thus attracting polar molecules (such as creatinine) better. Meanwhile, the creatinine clearance value of the PES/GO350 membranes indicates a higher ability for creatinine removal compared to the PES membranes; therefore, the percentage of creatinine clearance in PES/GO350 was lower than that in PES itself. This is in good agreement with the oxygen-containing groups of GO forming hydrogen bonds with molecules of creatinine making it easier to pass through the membrane. This result indicates that addition of GO into the membranes improved the membrane’s ability to remove creatinine molecules and it may be considered as a substitute material for hemodialysis membranes. Furthermore, comparison between PES/GO350 with the other GO membranes is also shown in Table 3. The data show that the increasing temperature for the synthesis of GO will decrease the flux. In general, addition of GO increases the flux of the membrane, with GO contributing its hydrophilic properties to the membrane. As mentioned for the Raman data above, however, the minimal hydrophilic regions on the higher temperature synthesised GO result in a lower flux value. In particular, the presence of hydrophobic regions on GO is also crucial for mediating the physical interactions between GO and PES.

## Conclusions

GO was successfully synthesized by a pyrolysis method and can be incorporated in PES MMMs, where in SEM images of the PES/GO membranes, GO particles were uniformly dispersed. Addition of GO into the membranes also improves the hydrophilicity and mechanical strength properties of the membranes. The dialysis performance of the PES/GO membranes indicated a better flux and solute clearance compared to those of the pure PES membranes. This result indicates that the PES/GO350 membranes could be considered as good candidates for hemodialysis membranes.

## Conflicts of interest

The authors declare there are no conflicts.

## Supplementary Material

RA-008-C7RA11247E-s001
