# Supplementary material for: Incorporation of graphene oxide in polyethersulfone mixed matrix membranes to enhance hemodialysis membrane performance
Source: RSC Adv. 2018 Jan 3;8(2):931–7. doi: 10.1039/c7ra11247e (PMC9077013; doi:10.1039/c7ra11247e)
Supplement: RA-008-C7RA11247E-s001 [file RA-008-C7RA11247E-s001.pdf]

Electronic Supplementary Material (ESI) for RSC Advances.
This journal is © The Royal Society of Chemistry 2017

Supporting Information

Incorporation of Graphene Oxide on Polyethersulfone Mixed Matrix Membrane to Enhance Hemodialysis Membrane Performance

M. Wathoniyyah,[a] M. Khasanah,[a] Y. Rahardjo,[a] S. Wafiroh,[a] Abdulloh,[a] and M. Z. Fahmi[ab]*

a)

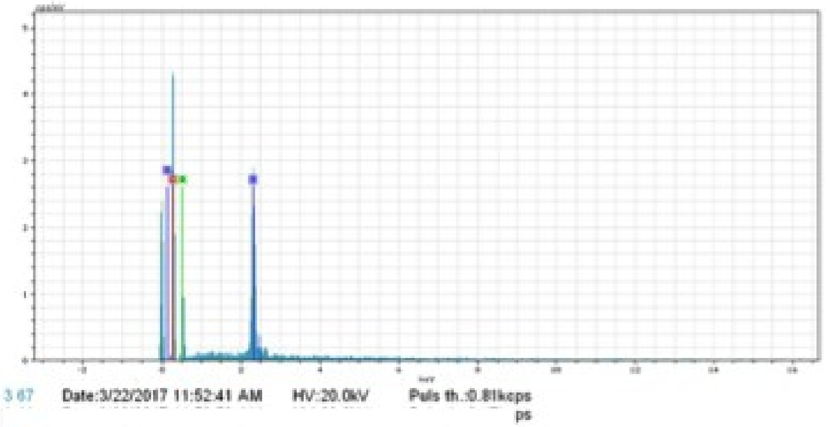

El	AN	Series	unn. C [wt.%]	norm. C [wt.%]	Atom. C [at.%]	Error [%]
C	6	K-series	44.30	44.30	55.16	15.3
O	8	K-series	39.80	39.80	34.17	19.4
S	16	K-series	15.90	15.90	10.67	8.5
		Total:	100.00	100.00	100.00	

b)

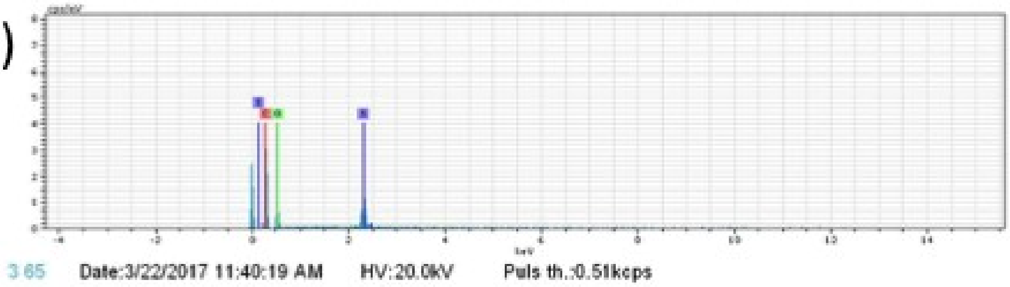

El	AN	Series	unn. C [wt.%]	norm. C [wt.%]	Atom. C [at.%]	Error [%]
C	6	K-series	48.49	48.49	58.06	17.1
O	8	K-series	41.83	41.83	37.60	16.8
S	16	K-series	9.68	9.68	4.34	0.5
		Total:	100.00	100.00	100.00	

Figure S1. Graph of EDX result on Figure 5 for (a) PES and (b) PES/GO.

The creatinine concentration was measured by colorimetric assay using picric acid as chromogenic agent. 2 ml mimic blood sample was mixed with 1 ml picric acid solution (26 mM) in 1 M NaOH solution and 1 ml pure water at 37 °C for 15 min. Then the UV absorbance of the complex compound reacted by creatinine and picric acid was detected using the UV–vis spectrophotometer at a wavelength of 510 nm, and the creatinine concentration in the sample was calculated according to a standard curve. At least three runs

were conducted for each sample so as to ensure reproducibility. Finally, the creatinine clearance was calculated by (5)

where are the concentrations of creatinine in the mimic blood before and after membrane dialysis.
